# Death from a Medico-Legal Aspect.—III

**Published:** 1911-12-02

**Authors:** 


### (ii) Death by Hanging.

The appearances found after death by hanging are not uniform or constant, and there is no single sign invariably present diagnostic of death by hanging. Indication of suspension, but not necessarily of death by such means, is the mark of the cord on the neck. The mark varies, however, in position and character according to the mode of suspension and the nature of the material of which the cord is made. The face is sometimes, but not commonly, distorted and expressive of suffering. Usually it is placid and pale, though after long suspension it may become livid. The eyes are sometimes very prominent, and the pupils are dilated. Frothy mucus may be found at the mouth and nostrils. The tongue is pressed against the teeth and indented, or it may be clenched between the jaws. The base of the tongue is injected. The hands are often tightly clenched, the nails being driven into the palms.

### Simulated Symptoms.

Two principal medico-legal questions arise in regard to persons found hanged: Did the suspension take place during life or after death? and was the hanging accidental, suicidal, or homicidal? In cases of hanging during life the cord does not always produce the same appearances. In some cases there is a well-marked bruise or ecchymosis, in others a pale indentation and a condensed state of the subcutaneous tissues resembling old parchment; in others, again, a hard chocolate-coloured groove; and these marks, limited to the fore part of the neck, may be combined at the angle of the jaw with a singed appearance. The cuticle may also be abraded here and there. Can the appearances occasioned by the cord during life be produced after death? This question has been answered in the affirmative. Bruises may be produced some time after life is extinct; and that which is true of bruises in general will of course hold good with respect to this particular form of bruise. Accordingly, Orfila proved, by experiments on the dead body, that up to eighteen hours after death precisely the same appearances may be produced as in suspension during life. Casper sums up the results of a long series of experiments by the remarkable statement "that any ligature with which any body may be suspended or strangled, not only within a few hours, but even days after death, especially if the body be forcibly pulled downwards, may produce a mark precisely similar to that which is observed in most of those hanged while alive"; and he adds that the mark of the cord is a cadaveric phenomenon.

The presumption in all cases of suspension is favourable to the idea of suicide, since hanging is a difficult mode of perpetrating murder unless the strength of the parties be greatly disproportionate, or the assailants be numerous and powerful. And, accordingly, we find that, in a vast majority of cases, it is an act of suicide. We should first ascertain whether suspension took place before or after death; and next, the immediate cause of death. The instrument of death—that is, the cord—should be compared with the furrow that it has made, so as to ascertain whether the diameter of the neck be much diminished by it. All the circumstances which indicate strangulation are so far against the idea of suicide.

### An Important Case.

Marc Antoine Calas was the son of John Calas, a merchant of Toulouse, aged seventy years, of great probity, and a Protestant. This son was twenty-eight years of age, of a robust habit, but melancholy turn of mind. He was a student of law, and, becoming irritated at the difficulties he experienced (in consequence of not being a Catholic) concerning his licence, he resolved to hang himself. This he effected by fastening the cord to a billet of wood placed on the folding doors which led from his father's shop to his store-room. Two hours after he was found lifeless. The parents unfortunately removed the cord from the body, and never exhibited it to show in what manner his death was accomplished. No examination was made. The people, stimulated by religious prejudice, carried the body to the town hall, where it was the next day examined by two medical men, who, without viewing the cord or the place where the death had been consummated, declared that he had been strangled. On the strength of this the father was condemned by the Parliament of Toulouse, in 1761, to be broken on the wheel. He expired with protestations to Heaven of his innocence. On reflection, however, when it was too late, it was recollected that the son had been of a melancholy turn of mind; that no noise had been heard in the house while the deed was doing; that his clothes were not in the least ruffled; that a single mark only was found from the cord, which indicated suspension by suicide; and, in addition to these, that the dress proper for the dead was found lying on the counter. Voltaire espoused the cause of the injured family, and attracted the eyes of all Europe to this judicial murder. The cause was carried up to the Council of State, who, on May 19, 1765, reversed the decree of Parliament and vindicated the memory of John Calas (Foderé, vol. iii. p. 167).

In strangulation, strictly considered, as distinguished from hanging, the murdered person is not suspended. It is a more common, and probably a more violent, mode of murder than hanging. There are instances on record where injuries have been inflicted on bodies strangled to avert suspicion of the true manner in which they were killed. A cord applied a few hours after death would not produce so much bruise as would result from its application during life, and the turgescence of the face and characteristic post-mortem appearances would be wanting. As a general rule, cases of accidental strangulation present no difficulty to a medical jurist, provided the relations of the body to surrounding objects and the compressing force have not been disturbed. The slight injuries caused by the suffocation of helpless people led to the selection of this mode of death by the persons engaged in the supply of bodies to the anatomical schools prior to the passing of the Anatomy Act.

